# Improving SAR Target Recognition Performance Using Multiple Preprocessing Techniques

**DOI:** 10.1155/2021/6572362

**Published:** 2021-08-04

**Authors:** Qinmin Ma

**Affiliations:** School of Artificial Intelligence, Shenzhen Polytechnic, Shenzhen 518055, China

## Abstract

The synthetic aperture radar (SAR) image preprocessing techniques and their impact on target recognition performance are researched. The performance of SAR target recognition is improved by composing a variety of preprocessing techniques. The preprocessing techniques achieve the effects of suppressing background redundancy and enhancing target characteristics by processing the size and gray distribution of the original SAR image, thereby improving the subsequent target recognition performance. In this study, image cropping, target segmentation, and image enhancement algorithms are used to preprocess the original SAR image, and the target recognition performance is effectively improved by combining the above three preprocessing techniques. On the basis of image enhancement, the monogenic signal is used for feature extraction and then the sparse representation-based classification (SRC) is used to complete the decision. The experiments are conveyed on the moving and stationary target acquisition and recognition (MSTAR) dataset, and the results prove that the combination of multiple preprocessing techniques can effectively improve the SAR target recognition performance.

## 1. Introduction

Synthetic aperture radar (SAR) is widely used in military and civilian fields because of its all-weather data measurement and imaging capabilities. The key technology represented by SAR automatic target recognition (ATR) has become an important support for intelligence reconnaissance, missile guidance, and other links [1]. With the development and maturity of high-resolution SAR imaging technology, tactical target recognition methods based on SAR images have emerged. These methods mainly adopt the two-stage idea of “feature extraction + classifier.” Feature extraction starts from the original SAR image and extracts valuable target features, such as geometric shape, gray-scale distribution, and scattering characteristics, based on the idea of removing the roughness and keeping the essence. In [2–7], the geometric shape features such as target area (shadow) and contour were used to design SAR target recognition methods, which reflected the physical appearance information of the target. However, due to common interference such as noise and clutter in SAR images, the precision of features such as target regions and contours is often limited. In [8–15], principal component analysis (PCA), monogenic signal, mode decomposition, and other mathematical projection or signal decomposition algorithms were employed to obtain SAR image features. Such features have good consistency and high extraction efficiency. The disadvantage is that they often fail to reflect the physical layer information of the target. The characteristics that describe the scattering characteristics of SAR targets include polarization characteristics and local scattering centers. At this stage, the most used scattering feature is the scattering center, and the decision is made through the matching of attribute parameters (position, length, structure, and so on) [16–18]. Since the scattering center model is generally very complicated, it is difficult to estimate the parameters of the scattering center with high efficiency and precision. The classifiers used in SAR target recognition are mostly inherited from the field of optical pattern recognition or optimized and improved according to the characteristics of SAR images, such as K nearest neighbors (K-NN) in [8], support vector machine (SVM) used in [19, 20], adaptive boosting [21], the sparse representation-based classification (SRC) [22–26], and the recently popular convolutional neural network (CNN) [27–41].

Different from general optical images, there are a lot of noise interference in SAR images, which often results in poor visibility and readability. Therefore, before carrying out target recognition, using certain preprocessing methods to process SAR images can effectively improve the subsequent recognition performance. Generally, the size and pixel processing of the original SAR image can be used to improve the SAR image quality so as to facilitate subsequent feature extraction and classifier design. In the existing literature, a large number of preprocessing techniques have been adopted and verified, including image cropping, target segmentation, image enhancement, and superresolution. In this paper, three types of preprocessing methods, image cropping, target segmentation, and image enhancement, are adopted for the problem of SAR image target recognition. On this basis, the monophonic signal is further used as the basic method of feature extraction to obtain multilevel spectrum features. Based on sparse representation classification, the results of preprocessing and feature extraction are classified, and the final decision result is obtained. Experiments are conveyed on the moving and stationary target acquisition and recognition (MSTAR) dataset. The results validate the effectiveness of the proposed method.

## 2. Description of Preprocessing Techniques

### 2.1. Image Cropping

Image cropping is a very common preprocessing technique in SAR target recognition, which can efficiently eliminate a large amount of background redundancy in original SAR image. The image cropping operation is very simple, by segmenting a square area with a certain side length in the center of the original SAR image as the target image. The selected side length of the square has a certain influence on the final target recognition performance. The larger the side length is, the more background clutter will be removed, but at the same time it is possible to remove a part of the target area. Therefore, it is very important to select a suitable cropping window. When the window is too small, the target characteristics are likely to be destroyed to a certain extent. On the opposite, a very large window may keep too many background and clutter pixels. As a result, those interferences still exist.

### 2.2. Target Segmentation

The purpose of target segmentation is to separate the target area from the background pixels and target shadows so as to eliminate the interference of background noise as much as possible. Compared with optical images, the visibility of SAR images is poor and the target boundary is not clear. Therefore, SAR target segmentation has always been a difficult problem, and it is difficult to reliably evaluate the performance of a certain target segmentation algorithm. In this paper, the target segmentation algorithm proposed in the literature is used, and the specific implementation steps are as follows:

 
*Step 1*. Performing histogram equalization on the original SAR image, and transforming its dynamic range to [0, 1]
 
*Step 2*, Performing average filtering on the histogram equalized image
 
*Step 3*. Using the threshold method for image segmentation, and the threshold value is 0.8
 
*Step 4*. Aiming at the influence of possible small fractures and cavities in the target area and background clutter, mathematical morphology operations are used to eliminate them

With the help of high-precision target segmentation, the pure target characteristics can be maintained while the interferences can be efficiently eliminated. In the next stage, the features can be extracted only in the target region so the effectiveness can be better maintained.

### 2.3. Image Enhancement

Image enhancement uses certain image processing technology to highlight some information in the image or weaken or eliminate some irrelevant information for the application requirements of certain characteristics. Therefore, it can enhance the ability to interpret the information of interest. For the specific application of SAR target recognition, a large number of image enhancement techniques have been adopted, such as contrast enhancement, image filtering, and power exponential enhancement. In this paper, power exponent enhancement is used to preprocess the original SAR image. The specific operation is as follows. First, the power transformation of the gray value of the original SAR image is as follows:

(1) $$\begin{array}{c} K\left(x,y\right)={\left[I\left(x,y\right)\right]}^{\alpha }. \end{array}$$

Then, the power-transformed pixel value is normalized according to the following equation:

(2) $$\begin{array}{c} J\left(x,y\right)=\frac{K\left(x,y\right)}{{\left(\sum_{x}\sum_{y}{\left|K\left(x,y\right)\right|}^{2}\right)}^{1/2}}. \end{array}$$

The enhancement effect under different powers is not the same. At a suitable power choice, the image enhancement has the effect of suppressing the background and enhancing the target characteristics, which is beneficial for the following feature extraction and correct target recognition.

## 3. Application of Target Recognition

### 3.1. Feature Extraction by Monogenic Signal

The monomorphic signal is a two-dimensional analytical signal that has the ability to analyze the two-dimensional time-frequency characteristics of the image so as to analyze the rich texture and detailed features of the target. At present, monophonic signals have been effectively used in face image and SAR image recognition [11, 12]. Denote *f*(*z*) as the 2D signal, and its Riesz transform is calculated as *f*_*R*_(*z*), where *z*=(*x*, *y*)^*T*^ denotes the 2D spatial domain coordinate. At first, the two-dimensional Riesz transform of the original signal is calculated as follows:

(3) $$\begin{array}{c} f_{M}\left(z\right)=f\left(z\right)-\left(i,j\right)f_{R}\left(z\right), \end{array}$$

where *i* and *j* are the imagery units. The original signal and Riesz transform comprise the real and imaginary parts of the monogenic signal. Then, three monogenic components, i.e., local amplitude, local phase, and local orientation, can be obtained as following equation:

(4) $$\begin{array}{c} \text{amplitude:}A\left(z\right)=\sqrt{f{\left(z\right)}^{2}+{\left|f_{R}\left(z\right)\right|}^{2}},\\ \text{phase:}\varphi \left(z\right)=a\;\tan\;2\left(\left|f_{R}\left(z\right)\right|,f\left(z\right)\right)\in \left(-\pi ,\pi \right],\\ \text{orientation:}\theta \left(z\right)=a\;\tan\;2\left(\frac{f_{y}\left(z\right)}{f_{x}\left(z\right)}\right)\in \left(-\frac{\pi }{2},\frac{\pi }{2}\right], \end{array}$$

where *f*_*x*_(*z*) and *f*_*y*_(*z*) are the *i*-imaginary and *j*-imaginary components, respectively.

The Riesz transformation and Log-Gabor in the decomposition process of the monophonic signal are both performed in two dimensions, so the decomposed *A*(*z*), *φ*(*z*), and *θ*(*z*) finally are in the form of a two-dimensional matrix consistent with the original image size. They have different characteristics and have the ability to describe the characteristics of the original image from different sides. The local amplitude focuses on reflecting the intensity (gray value) distribution of the image. The local phase and local orientation describe the local details of the image and the target shape information, respectively. Therefore, making full use of the multilevel spectral components obtained by the single-analysis signal decomposition is beneficial to describe the target characteristics more comprehensively, thereby improving the subsequent classification accuracy. With reference to the parameter settings in [11], this study defines 3 Log-Gabor filters of different scales so as to obtain 3 levels and 9-component monochromatic spectrum components, which are combined as one feature vector. It is validated that each spectral component at different levels can effectively reflect part of the characteristics of the original SAR image, but there is also a certain degree of redundancy. Therefore, it is necessary to effectively screen a large number of spectral components obtained by decomposition so as to comprehensively improve the accuracy and efficiency of subsequent classification.

### 3.2. SRC for Classification

The sparse representation is based on the theory of compressed sensing and analyzes the characteristics of the sample by linearly characterizing the sample with unknown characteristics on the overcomplete dictionary. Wright et al. used sparse representation in face recognition, that is, to determine the category of the test sample based on the reconstruction error of each category under the sparse representation coefficient [42, 43]. Specifically, a global dictionary *A*=[*A*_1_, *A*_2_,…, *A*_*C*_] ∈ R^*d*×*N*^ is first constructed composed of multiple training categories, where *A*_*i*_ represents the *N*_*i*_ atom corresponding to the *i*th training sample in the class. For the test sample *y* to be identified, the sparse linear representation is performed as follows:

(5) $$\begin{array}{c} \hat{x}=\underset{x}{\arg\min}{\left\|x\right\|}_{0},\\ \text{s.t. }{\left\|y-\text{Ax}\right\|}_{2}^{2}\leq \varepsilon , \end{array}$$

where *x* is the sparse coefficient vector to be solved and *ε* is the settled error threshold.

Since the direct solution of the optimization problem in equation (5) is very complicated, researchers obtain high-confidence approximate solutions through the principle of equivalent approximation. For example, in [42], the *ℓ*_1_ norm is used to replace the original *ℓ*_0_ norm to convert it into a convex optimization problem that is easy to solve. In [22], the orthogonal matching pursuit (OMP) algorithm was employed based on a greedy mechanism to improve the overall solution efficiency. According to the solved sparse representation sparse vector, the category of the test sample can be judged according to its distribution rules in different categories. Among many principles for decision, the criterion based on the minimum reconstruction error is the most widely used. The basic idea is to linearly reconstruct the test samples with samples of each category and then calculate the reconstruction error, as follows:

(6) $$\begin{array}{c} r\left(i\right)={\left\|y-A_{i}x_{i}\right\|}_{2}^{2}\;\left(i=1,2,\ldots ,C\right), \end{array}$$

where *x*_*i*_ includes the linear coefficients related to the *i*th training class and *r*(*i*) is the reconstruction error from *i*th training class. Finally, SRC makes the decision based on the least error.

### 3.3. Procedure of Implementation

**Figure 1:**
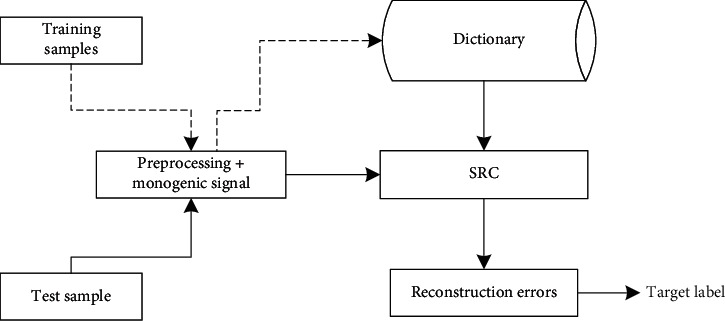
Procedure of the proposed method for target recognition.

Based on the above discussions, the basic procedure of the proposed method is summarized as shown in Figure 1. The training and test samples are first processed by the three preprocessing techniques. Afterwards, the monogenic signal is used to extract the features from the training samples to establish the global dictionary. The monogenic feature vector from the test sample is represented by the global dictionary, and the reconstruction errors from different classes are compared to determine the target class. Specially, the MSTAR SAR images for experiments are cropped to the size of 80 × 80 to intactly cover the target regions. Furthermore, the power factor used in image enhancement is chosen to be 2.5 to achieve a relatively good result.

## 4. Experiments

### 4.1. MSTAR Dataset

**Figure 2:**
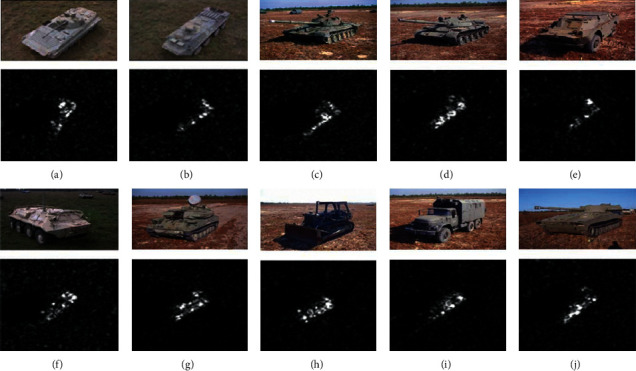
Imagery of targets in the MSTAR dataset to be recognized: (a) BMP2; (b) BTR70; (c) T72; (d) T62; (e) BRDM2; (f) BTR60; (g) ZSU24/4; (h) D7; (i) ZIL131; (j) 2SI.

Set the experimental conditions based on the MSTAR data set to carry out the classification experiment of multiclass targets. As a currently widely used SAR target image data set, MSTAR data contain 10 types of vehicle targets acquired under various conditions (see Figure 2 for examples of optical and SAR images). In these images, the target azimuth angle covers 0°∼360°, and some targets have several submodels (such as BMP2 and T72); the original image resolution reaches 0.3 m.  Table 1 shows one of the typical experimental conditions based on SAR images of 10 types of targets. Among them, the training and test sets use samples at the elevation angles of 17° and 15°, respectively; the test sets of BMP2 and T72 contain more submodels than the training set. Under the current setting conditions, the gap between training and testing samples is relatively small, which is generally approximate to standard operating conditions (SOCs).

In order to verify the effectiveness and robustness, the proposed method is compared with several reference methods from current literatures including the SRC-based method in [22], method using monogenic signal for feature extraction in [11], and method based on CNN in [30]. The following tests are conducted under both the standard operating condition (SOC) and extended operating conditions (EOCs) to achieve comprehensive evaluations on the proposed method.

### 4.2. Validation of Processing Techniques under SOC

**Figure 3:**
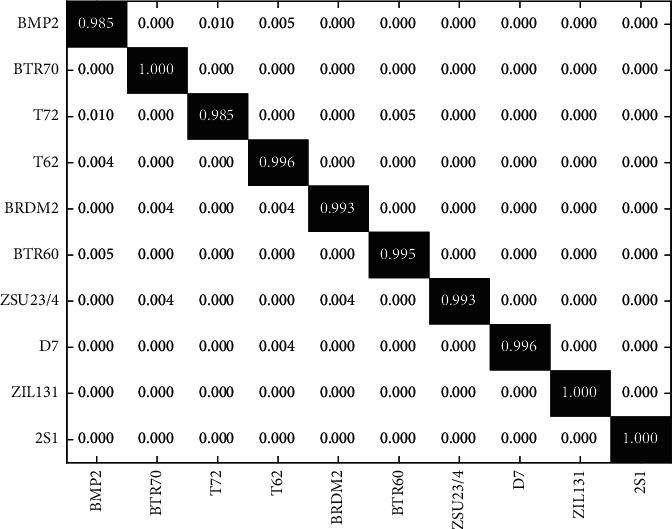
Results achieved by the proposed method under SOC.

A preliminary validation is conveyed under SOC, whose experimental setup is shown in Table 1. The training samples are those SAR images of the ten targets measured at 17° depression angle. The test samples are from 15° depression angle with extra target configurations in BMP2 and T72. The results achieved by the proposed method are displayed in Figure 3, in which the recognition accuracies of different targets are marked on the diagonal. BMP2 and T72 suffer the lower recognition rates than the remaining ones because of the disturbance caused by configuration variances.  Table 2 lists the average recognition rates of different methods. In particular, we compare the proposed method with the one without the three preprocessing techniques. The comparison shows that the use of preprocessing techniques effectively improves the recognition performance. Compared with the three reference methods, the proposed method achieves the highest result, validating its superior effectiveness.

### 4.3. Depression Angle Variances

In the experimental setup under SOC, the depression angles of the test and the training samples are very close (only 2° difference). In practice, the test sample is likely to be at a different depression angle from the training set. At this time, the image differences caused by the difference in the depression angle increase the difficulty of recognition.  Table 3 shows the experimental conditions with large depression angle differences. The samples at 17° depression angle are used to classify the test sets at 30° and 45°, respectively.  Table 4 comprehensively shows the average recognition rate of each method at the two depression angles. It can be clearly seen that when the depression angle is 45°, the performance of each method drops significantly. Comparing and analyzing under the two test conditions, the proposed method obtains the best performance, which shows its robustness to the change of depression angle. The preprocessing techniques could effectively improve the image quality even under the situation of depression angle variances. Then, the features after preprocessing can better handle the EOC caused by depression angle variances.

### 4.4. Noise Corruption

**Figure 4:**
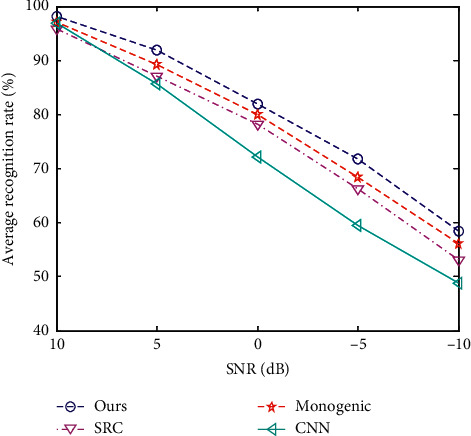
Average recognition rates at different SNRs.

Noise exists in the whole process of SAR data acquisition and imaging, so it is an important factor that must be considered in SAR target recognition. As the noise continues to intensify, the original target characteristics are continuously disturbed or even submerged. The images in the original MSTAR dataset are all collected from cooperative conditions, which are less affected by noise interference and have a higher signal-to-noise ratio (SNR). For this reason, this experiment implements noise addition to the test samples in Table 1 according to the idea in [17] and then uses the proposed and reference methods to classify the noise samples.  Figure 4 shows the performance curves of different methods. Through the preprocessing techniques, the noise interferences can be effectively relieved so the features in the following stage can maintain higher discrimination. Comparing all methods comprehensively, the method in this paper has the strongest noise robustness.

## 5. Conclusion

This paper discusses the influence of SAR image preprocessing technology on target recognition performance and analyzes and studies three preprocessing technologies of SAR image cropping, target segmentation, and image enhancement. The preprocessed SAR image is extracted from the features of the monomorphic signal and classified based on the SRC to obtain the target category of the test sample. Experiments on the MSTAR dataset are carried out. It can be seen from the experimental results that the recognition rate obtained after preprocessing is significantly improved. At the same time, the method is also more robust to EOCs such as depression angle differences and noise interference.

## Figures and Tables

**Table 1:** Description of training and test samples under SOC.

Target class	Training (17°)		Test (15°)	
	Configuration	Number of samples	Configuration	Number of samples
BMP2	9563	222	9563	184
			9566	185
			c21	185

BTR70	c71	222	c71	185

T72	132	221	132	185
			812	184
			s7	180

T62	A51	288	A51	262
BRDM2	E-71	287	E-71	263
BTR60	7532	245	7532	184
ZSU23/4	d08	288	d08	263
D7	13015	288	13015	263
ZIL131	E12	288	E12	263
2S1	B01	288	B01	263

**Table 2:** Results comparison under SOC.

Method type	Average recognition rate (%)
Ours	99.02
Ours without preprocessing	97.64
SRC	97.18
Monogenic	98.06
CNN	98.85

**Table 3:** Experimental setup under depression angle variances.

Class	Configuration	Depression angle		
		Training	Test	
		17°	30°	45°
2S1	B01	288	277	292
BRDM2	E-71	287	276	292
ZSU23/4	d08	288	277	292

**Table 4:** Average recognition rates of different methods under depression angle variances.

Recognition method		Ours	SRC	Monogenic	CNN
Average recognition rate (%)	30°	97.28	95.68	96.82	96.34
	45°	72.06	68.74	70.26	68.15

## Data Availability

The MSTAR dataset is publicly available.
